# Gene–Gene and Gene-Sex Epistatic Interactions of *MiR146a*, *IRF5*, *IKZF1*, *ETS1* and *IL21* in Systemic Lupus Erythematosus

**DOI:** 10.1371/journal.pone.0051090

**Published:** 2012-12-07

**Authors:** Rui-Xue Leng, Wei Wang, Han Cen, Mo Zhou, Chen-Chen Feng, Yan Zhu, Xiao-Ke Yang, Mei Yang, Yu Zhai, Bao-Zhu Li, Xiao-Song Wang, Rui Li, Gui-Mei Chen, Hong Chen, Hai-Feng Pan, Dong-Qing Ye

**Affiliations:** 1 Department of Epidemiology and Biostatistics, School of Public Health, Anhui Medical University, Hefei, Anhui, People’s Republic of China; 2 Anhui Provincial Laboratory of Population Health & Major Disease Screening and Diagnosis, Anhui Medical University, Hefei, Anhui, People’s Republic of China; University of Michigan Medical School, United States of America

## Abstract

Several confirmed genetic susceptibility loci involved in the interferon signaling and Th17/B cell response for SLE in Chinese Han populations have been described. Available data also indicate that sex-specific genetic differences contribute to SLE susceptibility. The aim of this study was to test for gene–gene/gene-sex epistasis (interactions) in these known lupus susceptibility loci. Six single-nucleotide polymorphisms (SNPs) in *MiR146a*, *IRF5*, *IKZF1*, *ETS1* and *IL21* were genotyped by Sequenom MassArray system. A total of 1,825 subjects (858 SLE patients and 967 controls) were included in the final analysis. Epistasis was tested by additive model, multiplicative model and multifactor dimensionality reduction (MDR) method. Additive interaction analysis revealed interactions between *IRF5* and *IKZF1* (OR 2.26, 95% CI 1.48–3.44 [P = 1.21×10^4^]). A similar tendency was also observed between *IL21* and *ETS1* by parametric methods. In addition, multiple high dimensional gene-gene or gene-sex interactions (three-and four-way) were identified by MDR analysis. Our study identified novel gene–gene/gene-sex interactions in lupus. Furthermore, these findings highlight sex, interferon pathway, and Th17/B cells as important contributors to the pathogenesis of SLE.

## Introduction

Systemic lupus erythematosus (SLE) is a prototypic, systemic, autoimmune disease, characterized by a diverse array of autoantibody production, complement activation and immune-complex deposition, which causes tissue and organ damage. The aetiology of SLE is incompletely understood, but genetic factors play an important role in the susceptibility to the disease.

Recent candidate gene and genome-wide association studies (GWAS) led to the discovery and validation of multiple susceptibility loci for SLE. The loci previously confirmed for SLE in Chinese include genes involved in the interferon signaling (eg. *IRF5*, *IRF7*, *Mir146a*, *IKZF1*) and Th17/B cell regulation (eg. *ETS1*) [1]–[3]. The reported effect of IL21 on B-cell differentiation into plasma cells and its effect on Th17-cell responses make *IL21* an attractive candidate gene for SLE [4]. Previous studies established and confirmed the genetic association between *IL21* and lupus in European descent [4]–[5]. In a recent case-control study (605 patients, 666 controls), Ding *et al* showed that polymorphisms of *IL21* gene have a marginal association with SLE susceptibility in the Chinese populations [6]. Most above pathway genes are known to play a key role in the pathogenesis of the disease. Since the heritability of SLE cannot be completely explained by the susceptibility loci already discovered. Herein, we sought to examine gene–gene interactions (epistasis) in some of the previously established susceptibility loci for SLE in Chinese populations.

Current data also indicate that sex-specific genetic differences contribute to SLE susceptibility. For example, the frequency of the risk alleles in the *HLA*, *IRF5* and osteopontin (*SPP1*) locus was significantly higher in men than in women with SLE [7], [8]. Interestingly, polymorphism in the *KIAA1542* locus was shown to be associated with lupus in women but not in men [7]. Therefore, we also investigated gene–sex interactions in above genes.

## Results

### Replication of Genetic Association with SLE in Chinese

After the quality control measures were applied, a total of 1,825 subjects (858 SLE patients and 967 controls) were included in the analysis. Table S1 shows demographic characteristics for study participants. The result of allelic association for single SNP is showed in Table 1. All SNPs in controls were under the Hardy-Weinberg equilibrium (HWE) (Table 1). In current study, 3 genes (*ETS1*, *IKZF1* and *IRF5*) previously reported in GWAS of Chinese Han populations were replicated. The SNP rs907715 in *IL21* showed association (P = 0.01) with SLE in Chinese. Association analysis using the genotype data (adjust for sex and age) generated a more significant result (P = 0.004). In the current study, the power to detect a 1.3-fold increased risk, assuming an alpha value of 0.05, was 0.997 for rs907715. However, significant association with SLE was not observed in the selected SNP for *MiR146a*.

**Table 1:** Genotype characteristics of each single nucleotide polymorphism.

Gene	SNP	Function	Allele^a^	Case (MAF)	Control (MAF)	OR (95% CI)	P-value	HWE^b^
*IL21*	rs907715	Intron	A/G	0.420	0.463	0.84 (0.74–0.96)	0.01	0.37
*IL21*	rs2221903	Intron	G/A	0.115	0.098	1.19 (0.96–1.47)	0.11	0.18
*IRF5*	rs4728142	Flanking 5' UTR	A/G	0.164	0.119	1.45 (1.20–1.75)	9.79×10^−5^	0.92
*IKZF1*	rs4917014	Flanking 5' UTR	G/T	0.267	0.331	0.74 (0.64–0.85)	2.56×10^−5^	0.39
*ETS1*	rs6590330	Flanking 3' UTR	A/G	0.407	0.345	1.30 (1.14–1.49)	1.15×10^−4^	0.26
*MiR146a*	rs57095329	Promoter	G/A	0.214	0.200	1.09 (0.93–1.28)	0.29	0.62

MAF, minor allele frequency; 95% CI, 95% confidence intervals; HWE, Hardy-Weinberg Equilibrium.

a nor allele/major allele;

b P-value for HWE.

### Additive Gene-gene Interactions Analysis by Direct Counting and Chi-square Test using a 2×2 Factorial Design

Because the numbers for some genotypes were small, and in most cases dominant models showed the better fit for association, we considered the dominant model in a further interaction analysis stage. In the current study, differences in risk genotype counts between patients and controls were high, which was particularly significant when risk genotypes were combined (Table S2). A significant additive interaction was observed between *IRF5* GA+AA and *IKZF1* GT+TT (OR 2.26, 95% CI 1.48–3.44 [P = 1.21×10^4^]); the risk genotype combination contributed the most to the overall interaction, with the remaining combinations within being nonsignificant. The attributable proportion due to interaction (AP) was 0.41, and the relative excess risk due to interaction (RERI) was 0.93. A similar tendency was also observed between *IL21* rs907715 AG+GG and *IRF5* GA+AA (OR 2.03, 95% CI 1.49–2.78 [8.13×10^6^] AP 0.14, RERI 0.29); *IL21* rs907715 AG+GG and *ETS1* GA+AA (OR 1.79, 95% CI 1.22–2.63 [P = 2.58×10^3^] AP 0.34, RERI 0.60); *IKZF1* GT+TT and *ETS1* GA+AA (OR 2.10, 95% CI 1.23–3.57 [P = 5.25×10^3^] AP 0.29, RERI 0.61).

### Multiplicative Interaction Effect Analysis by Logistic Regression

We tested whether the log likelihood of the logistic model was significantly improved by adding an additional pairwise interaction term for the combined 2 SNPs. The result of genetic interactions for pairwise SNP is showed in Table 2. A marginal effect of gene-gene interaction was detected between *ETS1* and *IL21* rs2221903 in codominant model (β = 0.26, P = 0.02). Dominant model also showed a consistent tendency toward a gene–gene interaction between *ETS1* and *IL21* rs907715 (β = 0.58, P = 0.03). We have not observed any genetic interactions for other SNP combinations by the codominant, dominant and recessive models (P>0.05).

**Table 2:** Interaction analysis of gene-gene involved in systemic lupus erythematosus, by logistic regression*.

Gene	*IL21*(rs907715)	*IL21*(rs2221903)	*IRF5*	*IKZF1*	*ETS1*
*IL21*(rs907715)					
*IL21*(rs2221903)	0.84/0.24				
*IRF5*	0.62/0.73	0.49/0.74			
*IKZF1*	0.27/0.09	0.09/0.72	0.34/0.38		
*ETS1*	0.25/**0.03**	**0.02**/0.17	0.49/0.83	0.92/0.50	
*Mir146a*	0.45/0.37	0.51/0.65	0.86/0.72	0.37/0.27	0.15/0.30

* The data are presented as P values (codominant/dominant) for departure from a multiplicative interaction model, obtained by log-likelihood ratio tests between the models, with and without an interaction term. An interaction term was considered significant if P<0.05. The regression models were adjusted for the potential confounders, including age and sex.

The result of pairwise gene-sex epistasis by logistic regression models is showed in Table S3. A marginal effect was detected between sex and *IL21* rs907715 in dominant model (β = 0.70, P = 0.03). We have not observed any multiplicative interactions for other gene-sex combinations by the codominant, dominant and recessive models (P>0.05). In order to corroborate the gene-sex interaction, we then compared risk allele frequencies between men and women with SLE (mean age was 35.75 years for male patients and 35.84 for female). There were no significant deviations in male-female frequencies (Table S4).

### Gene-gene and Gene-sex Interaction by MDR

Next, we used MDR analysis to identify potential high dimensional gene-gene interactions. The two- to four-way gene-gene interaction models are listed in Table 3. The SNP in the *ETS1* gene had the highest testing-balanced accuracy among the 6 SNPs. The model of *IL21* rs907715and *ETS1* showed the highest testing-balanced accuracy in two-way level (Figure S1a). The pairwise interaction was then validated based on 1000 permutations (p<0.03). A three-way interaction found between *IL21* rs907715, *IKZF1* and *ETS1* showed the highest testing-balanced accuracy and cross validation consistency in original data. Since our dataset is unbalanced, with 858 cases and 967 controls. An over- and under-sampling approach was applied to evaluate the high dimensional interaction. Based on the highest testing-balanced accuracy and cross validation consistency, a four-locus model (*IL21* rs907715, *IRF5, IKZF1, ETS1*) was chosen as the final model (Table S5).

**Table 3:** Summary of MDR gene-gene interaction results.

Model	Training Bal. Acc. (%)	Testing Bal. Acc. (%)	Cross-validation Consistency
*ETS1*	55.07	54.24	9/10
*IL21(rs907715), ETS1*	56.47	53.69	7/10
*IL21(rs907715), IKZF1,ETS1*	58.43	55.84	9/10
*IL21(rs907715), IRF5, IKZF1,ETS1*	60.19	55.72	9/10

Regarding high dimensional gene-sex interaction, a three-way interaction found between *IRF5, ETS1* and sex showed the highest testing-balanced accuracy and cross validation consistency in original data (Table 4, also see Figure S1b). The finding was further validated by over- and under-sampling approach (Table S6). In order to elucidate potential three-way gene-sex interactions in SLE, the top five three-way interaction models were listed (Table S7). The rank was determined by the training-balanced accuracy of MDR.

**Table 4:** Summary of MDR gene-sex interaction results.

Model	Training Bal. Acc. (%)	Testing Bal. Acc. (%)	Cross-validation Consistency
*Sex*	60.54	60.54	10/10
*Sex, IKZF1*	61.25	58.32	5/10
*Sex,IRF5, ETS1*	62.62	61.89	9/10
*Sex, IKZF1, ETS1, IL21(rs907715)*	63.51	59.07	5/10

## Discussion

Polymorphisms in *IRF5*, *IKZF1* and *ETS1* genes have been identified by recent GWAS in Chinese Han populations [1]. A functional variant (rs57095329) in *MiR146a* promoter also was found in 7,182 Asians by candidate gene approach [3]. The lupus-susceptibility gene *IL21* rs907715, identified in European descent [4]–[5], was replicated in current study. All these finding highlight that interferon signaling and Th17/B cell responses play an important role in the pathogenesis of SLE. Since previous studies have shown that polymorphisms in IFN pathway genes were especially associated with male SLE [7]–[10]. We then compared risk allele frequencies between men and women with SLE. The results showed that there was no significant difference in the patient group between men and women. It is noteworthy that the frequency of risk allele in the *IRF5* locus was significantly higher in men than in women with SLE in European descent [7]. In contrast, our data have not found a sex-specific genetic effect in *IRF5* rs4728142 (OR male-female = 1.06, P = 0.80), which previously were shown to be most significantly associated with SLE in Chinese [1].

It is well know that the best methodology for detecting interaction remains controversial. Combining several analytical methods may be optimal for detecting epistasis [11], [12]. To test for gene–gene/sex interactions, we performed a 2-step analysis using a parametric approach, followed by a nonparametric analysis. Since available data indicate that gene-gene interactions may occur between different pathophysiological pathways [12], [13], we examined gene-gene interactions for each SNP pair of the 5 genes. The data showed that there were multiple tendencies toward interactions in above genes.

Ikaros family zinc finger 1, encoded by *IKZF1*, is lymphoid-restricted zinc finger transcription factors. Recently data has suggested that IKZF1 has an important role in the induction of Type I interferon [14]. In current study, we showed evidence for gene–gene interaction between the 2 independent lupus-associated SNPs within the *IRF5* and *IKZF1* (AP = 0.41, RERI = 0.93). This interaction emphasizes the role of the interferon pathway in the pathogenesis of lupus. Moreover, Fang *et al* recently identified the IRF-5-Ikaros axis as a critical modulator of the B-cell immunoglobulin (Ig) G2a/c class switching. They reported that IRF5 can control expression of IKZF1. Mechanism analysis further showed that IRF8 activates the IKZF1 promoter, and IRF-5 inhibits the transcriptional activity of IRF-8 [15].

The gene-gene interaction between the *IL21* and *ETS1* was identified in both additive and multiplicative model. This finding is very interesting, because it highlights a critical role for Th17 and B cells in lupus. IL21 play an essential role for the promotion of B-cell and Th17 activation and differentiation [4]. Available data has shown that patients with SLE have increased plasma IL21 levels and proportion of IL21^+^ T-cells [16], [17]. In contrast, ETS1 is a negative regulator for both pathogenic B cell and Th17 cell responses [18]. ETS1 is critical in maintaining B cell identity and its deficiency can drive terminal differentiation of B cells into Ig-secreting plasma cells [19], [20]. Furthermore, ETS1-deficient Th cells differentiated more efficiently to Th17 cells than wild-type cells [21]. It is of note that the marginal effect (multiplicative interaction between *IL21* and *ETS1*) needs further replication on independent cohorts of SLE patients in order to be confident in the robustness of the results.

Except for the above interactions (*IRF5* and *IKZF1*, *IL21* and *ETS1*), we also identified additive interaction tendency between *IL21* rs907715 and *IRF5; IKZF1* and *ETS1.* Intriguingly, each of the four loci (*IL21*, *IRF5*, *IKZF1* and *ETS1)* can be mapped into B cell signaling pathways [4], [14], [15], [18]–[20]. In addition, previous study reported that ETS1 levels can strongly affect *MiR146a* promoter activity in vitro. The authors also observed additive effects of the risk alleles of *MiR146a* rs57095329 and *ETS1* rs1128334 [3]. Therefore, we further investigated whether there is an interaction between the risk variants of the two genes, rs6590330 in *ETS1* and rs57095329 in *MiR146a*. No significant epistatic effect was detected between the two variants in any additive and multiplicative models.

To strengthen the reliability of the interactions observed in current study, we conducted functional analysis by method of bioinformatics. We assessed relational biological network (pathway analysis, see Figure S2) constructed using Pathway Studio Explore Affymetrix Edition Version 1.1. However, the protein-protein interact and transcription factors targets prediction analysis did not find any directly associations (data not shown). For further explain for biological significance of interactions, we provided information of location for these SNPs (Table 1). Moreover, we also searched expression quantitative trait locus (eQTL) information from public database. The date showed that rs4728142 is an eQTL for *IRF5* in Asians (CHB, Han Chinese in Beijing/JPT, Japanese in Tokyo). In addition, the genotype of rs57095329 is directly related to expression of *MiR146a* expression [3]. However, the eQTL information of SNPs in *IL21*, *ETS1* and *IKZF1* is not available from public database. It is noteworthy that rs1128334 (located at the 3′-UTR region of *ETS1*) have an effect on the expression level of *ETS1*. Intriguingly, the SNP have high linkage disequilibrium (LD) with *ETS1* rs6590330 (r^2^ = 0.97) [22]. Nevertheless, further studies are required to comprehensively explore whether these SNPs is functional.

For two-way gene-sex interaction, we only detected a significant epistatic effect between sex and *IL21* rs907715 by logistic regression. However, we did not observe deviations in male-female frequencies that could corroborate their gene-sex interactions. An interaction between sex and *IRF5* locus, which was previously identified in patients with SLE of European descent [4], was not replicated in current study. It is of note that several limitations should be considered when interpreting the results of gene-sex interactions: First of all, the sample size of male patients with SLE is relatively small and is therefore of limited power, which may weaken the conclusion of this study. Secondly, it is unclear whether male SLE has the same disease severity or clinical features as female SLE. This question may be important because a more severe phenotype in lupus patients is often associated with higher total genetic risk [7].

MDR is a model-free method that can identify high dimensional gene-gene or gene-environment interactions [23], [24]. We applied the non-parametrical approach and found many three-and four-way gene-gene and gene-sex epistasis on SLE. These findings may account for some of the “missing heritability” in GWAS or candidate gene–based case–control studies for complex diseases. Furthermore, the data emphasize sex-specific genetic effects contribute to SLE susceptibility. It should be noted that the end goal of the MDR analysis is hypothesis generation. Therefore, the approach may be preferred to reduce the risk of false negatives [25].The nonparametric analysis of this study reflects a joint effect consisting of the main genetic effect and the interaction effect.

In current study, we observed that the combined effect of 2 risk factors would differ from the sum of the effects of the individual factors. Moreover, we provided possible evidence that in patients with lupus, the presence of one risk allele can influence the presence or absence of other risk alleles, across different loci (marginal association for multiplicative interaction). In addition, gender may also have an important influence for genetic risk of SLE. Taken together, our study identified novel gene–gene/gene-sex epistatic effect in lupus. Furthermore, these findings highlight sex, the interferon pathway, and Th17/B cells as important contributors to the pathogenesis of SLE.

## Materials and Methods

### Ethics Statement

The study was approved by the medical ethics committee of Anhui Medical University and complied with the principles outlined in the Helsinki Declaration. All participants gave written informed consent.

### Patients and Controls

Patients with SLE were recruited from the First Affiliated Hospital of Anhui Medical University and Anhui Provincial Hospital. The diagnosis of SLE was based on the presence of the combination of at least four criteria of 1997 American College of Rheumatology (ACR) revised criteria for the classification of SLE [26], [27]. The healthy control subjects were matched to the patients geographically and ethnically.

### SNPs Selection and Genotyping

SNP rs4728142 for *IRF5*, rs4917014 for *IKZF1* and rs6590330 for *ETS1*, which previously were shown to be most significantly associated with SLE in GWAS of Chinese Han populations [1], were selected for current case–control study. A novel Asian gene variant (rs57095329) in the promoter region of *MiR146a* was also selected [3]. Since previous study showed that polymorphisms of *IL21* gene have a marginal association with SLE susceptibility in the Chinese populations, we selected SNPs rs907715 and rs2221903 for *IL-21* genotyping [6]. *IRF7* rs1131665 is also a risk factor for the development of SLE in multiple populations including Chinese. However, the SNP was excluded in view of low allele frequency (MAF<5%) [2]. A total of 6 SNPs were included for further analysis (Table 1).

The genotyping was conducted using the Sequenom Massarray system (Sequenom, Inc., San Diego, CA, USA) according to the manufacturer’s instructions to determine the genotypes. Only those individuals with 100% genotype success for all markers were included for final analysis.

### Statistical Analysis

The genotype frequencies of the SNPs were tested for Hardy-Weinberg equilibrium in control subjects. Disease associations were analyzed by chi-square tests or by logistic regression analysis. Statistical power was estimated using Power and Sample Size Estimation Software (http://biostat.mc.vanderbilt.edu/wiki/Main/PowerSampleSize).

To test for additive gene-gene interactions, direct counting and chi-square tests were performed using a 2×2 factorial design to calculate the AP and RERI. Fisher’s exact test was used when necessary. If there is no biologic interaction, RERI and AP are equal to 0 [11].

The multiplicative interactions of two-way gene-gene and gene-sex were estimated using multiple logistic regression models. For each individual, key variables were defined as a binary variable indicating case–control status, with SNP variables ranging from 0 to 2 indicating the number of risk alleles in an individual subject (Codominant model). Additionally, dominant and recessive models for gene were also tested [28]. For each SNP pair, a logistic regression model was built to predict case–control status (dependent variable) based on the indicator variables (sex and age) and the 2 SNP variables (independent variable), for a total of 4 variables and an intercept [11]. For two-way gene-sex interaction analysis, age was used as indicator variable. We tested whether the log likelihood of the model was significantly improved by adding an additional pairwise interaction term [11]. SPSS11.0 software was applied for the logistic regression analysis. Two-tailed P values less than 0.05 were considered significant.

Multifactor dimensionality reduction software (MDR; http://sourceforge.net/projects/mdr/) is a model-free and non-parametrical approach method [23]. Since previous studies have shown MDR to be a useful method for identifying high dimensional gene-gene or gene-environment interactions [24]. We then applied MDR analysis to detect three-and four-way gene-gene/sex epistasis on SLE. For those interaction models that showed higher testing-balanced accuracy, we further used permutation tests to validate the MDR interaction results [25]. Since our dataset is unbalanced, with 858 cases and 967 controls. An over- and under-sampling approach was applied to evaluate non-parametrical interaction effect [25]. A brief description of the MDR approach and of the meaning of its output has been shown in others papers [13], [23], [25].

## Supporting Information

Figure S1 The optimal models as determined by MDR for *IL21* rs907715 and *ETS1* (a), Sex/*IRF5*/*ETS1* (b). (For SNP: 0 = no risk alleles, 1 = 1 risk allele, 2 = 2 risk alleles; For Sex: 1 = male, 2 = female). The numbers within each small square represent number of cases (left) and controls (right). For each square, dark-shading indicates high risk of disease, whereas light shading represents low risk of disease.
(DOC)

Figure S2 Relational gene network constructed using Pathway Studio Explore Affymetrix Edition Version 1.1 (a); Pathway Studio Explore Affymetrix Edition Version 1.1 was used to find linkages between 4 genes and diseases/cell biological processes (b).
(DOC)

Table S1 Characteristics of the patients and healthy controls studied.
(DOC)

Table S2 Additive interaction analysis of genes involved in SLE in genotype combinations by chi-square test using 2×2 factorial design.
(DOC)

Table S3 Interaction analysis of gene-sex involved in systemic lupus erythematosus, by logistic regression.
(DOC)

Table S4 Sex–gene disparities between men and women with systemic lupus erythematosus.
(DOC)

Table S5 Analysis of over- and under-sample data for gene-gene interaction.
(DOC)

Table S6 Analysis of over- and under-sampled data for gene-sex interaction.
(DOC)

Table S7 Three-way gene-sex interactions of MDR analysis.
(DOC)
