# Supplementary material for: Gene–Gene and Gene-Sex Epistatic Interactions of MiR146a, IRF5, IKZF1, ETS1 and IL21 in Systemic Lupus Erythematosus
Source: PLoS One. 2012 Dec 7;7(12):e51090. doi: 10.1371/journal.pone.0051090 (PMC3517573; doi:10.1371/journal.pone.0051090)
Supplement: Figure S1 — The optimal models as determined by MDR for IL21 rs907715 and ETS1 (a), Sex/IRF5/ETS1 (b). (For SNP: 0 = no risk alleles, 1 = 1 risk allele, 2 = 2 risk alleles; For Sex: 1 = male, 2 = female). The numbers within each small square represent number of cases (left) and controls (right). For each square, dark-shading indicates high risk of disease, whereas light shading represents low risk of disease. (DOC) [file pone.0051090.s001.doc]

**Figure S1: The optimal models as determined by MDR for *IL21* rs907715 and *ETS1* (a), Sex/*IRF5*/*ETS1* (b). (For SNP: 0= no risk alleles, 1= 1 risk allele, 2= 2 risk alleles; For Sex: 1= male, 2=female). The numbers within each small square represent number of cases (left) and controls (right). For each square, dark-shading indicates high risk of disease, whereas light shading represents low risk of disease.**

**(a).**

**
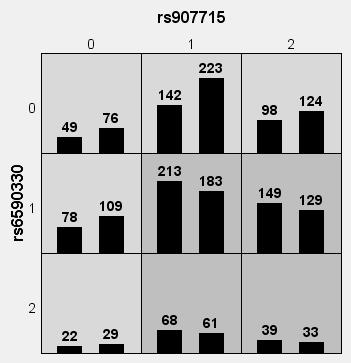
**

**(b).**

**
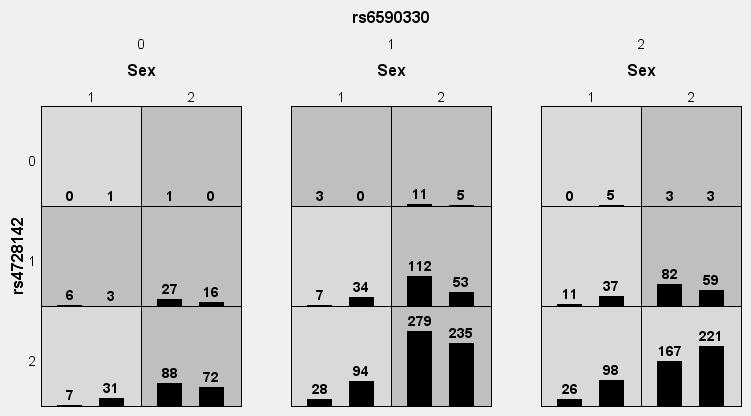
**
