# Supplementary material for: Gene–Gene and Gene-Sex Epistatic Interactions of MiR146a, IRF5, IKZF1, ETS1 and IL21 in Systemic Lupus Erythematosus
Source: PLoS One. 2012 Dec 7;7(12):e51090. doi: 10.1371/journal.pone.0051090 (PMC3517573; doi:10.1371/journal.pone.0051090)
Supplement: Figure S2 — Relational gene network constructed using Pathway Studio Explore Affymetrix Edition Version 1.1 (a); Pathway Studio Explore Affymetrix Edition Version 1.1 was used to find linkages between 4 genes and diseases/cell biological processes (b). (DOC) [file pone.0051090.s002.doc]

**Figure S2a. Relational gene network constructed using Pathway Studio Explore Affymetrix Edition Version 1.1**

**
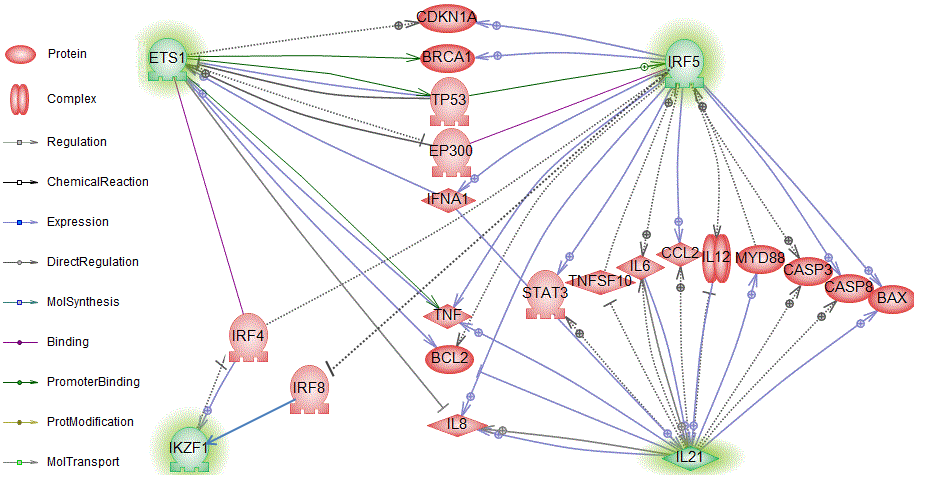
**

**
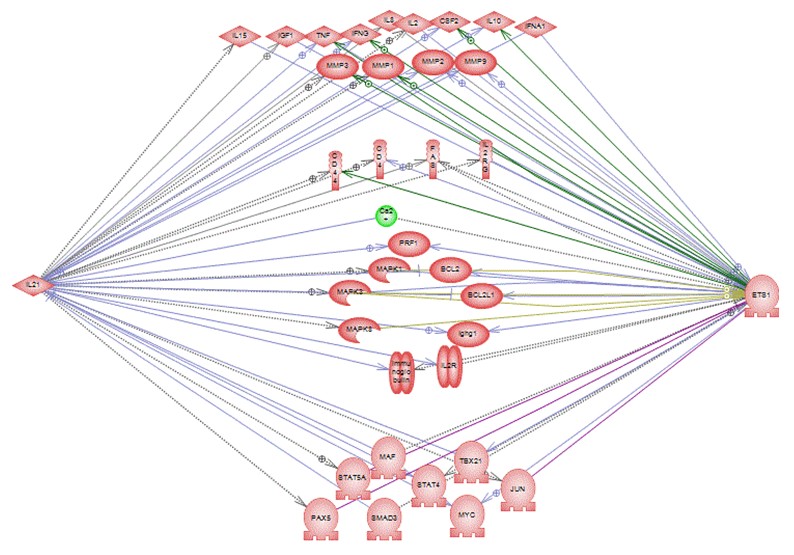
**

**Figure S2b.**

**Pathway Studio Explore Affymetrix Edition Version 1.1 was used to find linkages between 4 genes and diseases/cell biological processes.**

**
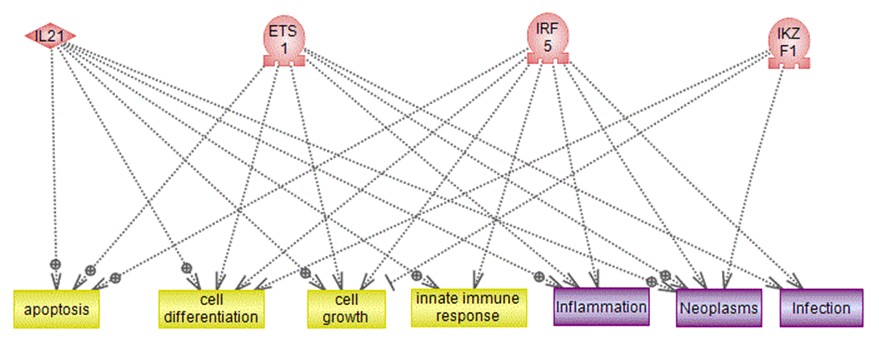
**

**
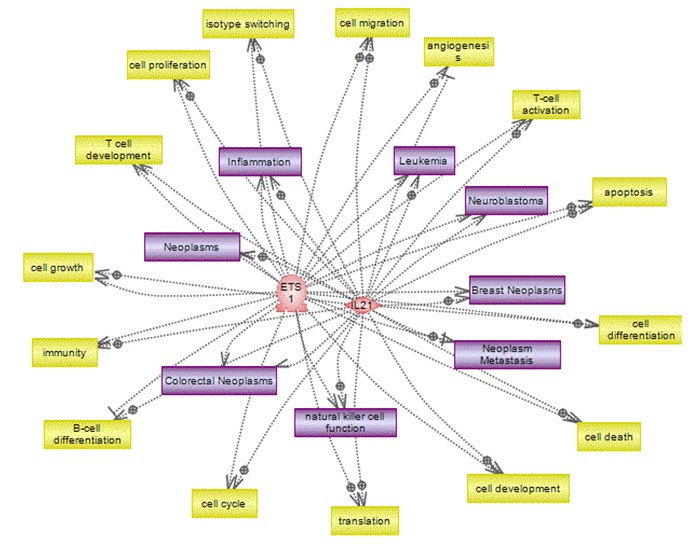
**
