# Supplementary material for: Gene–Gene and Gene-Sex Epistatic Interactions of MiR146a, IRF5, IKZF1, ETS1 and IL21 in Systemic Lupus Erythematosus
Source: PLoS One. 2012 Dec 7;7(12):e51090. doi: 10.1371/journal.pone.0051090 (PMC3517573; doi:10.1371/journal.pone.0051090)
Supplement: Table S1 — Characteristics of the patients and healthy controls studied. (DOC) [file pone.0051090.s003.doc]

**Table S1.** **Characteristics of the patients and healthy controls studied.**

|  | **SLE** | **Healthy controls** |
| --- | --- | --- |
| n | 858 | 967 |
| Male/Female | 88/770 | 303/664 |
| Age | 35.83±0.44 | 32.39±0.38 |

Values were presented by n or mean±SD.
