# Supplementary material for: Gene–Gene and Gene-Sex Epistatic Interactions of MiR146a, IRF5, IKZF1, ETS1 and IL21 in Systemic Lupus Erythematosus
Source: PLoS One. 2012 Dec 7;7(12):e51090. doi: 10.1371/journal.pone.0051090 (PMC3517573; doi:10.1371/journal.pone.0051090)
Supplement: Table S2 — Additive interaction analysis of genes involved in SLE in genotype combinations by chi-square test using 2×2 factorial design. (DOC) [file pone.0051090.s004.doc]

**Table S2** Additive interaction analysis of genes involved in SLE in genotype combinations by chi-square test using 2×2 factorial design

| **Combinations** | **SLE** | **Controls** | ***p _vs. con_*** | **OR (95% CI)** |
| --- | --- | --- | --- | --- |
| *IL21(rs907715)*/*IL21(rs2221903)*-0/0 | 149 | 214 |  | 1.00 |
| *IL21(rs907715)*/*IL21(rs2221903)*-0/1 | 0 | 0 |  |  |
| *IL21(rs907715)*/*IL21(rs2221903)*-1/0 | 521 | 576 | 0.03 | 1.30 (1.02-1.65) |
| *IL21(rs907715)*/*IL21(rs2221903)*-1/1 | 188 | 177 | 4.65×10^3^ | 1.53 (1.14-2.05) |
| *IL21(rs907715)*/*IRF5*-0/0 | 108 | 169 |  | 1.00 |
| *IL21(rs907715)*/*IRF5*-0/1 | 41 | 45 | 0.15 | 1.43 (0.88-2.32) |
| *IL21(rs907715)*/*IRF5*-1/0 | 487 | 582 | 0.05 | 1.31 (1.00-1.72) |
| *IL21(rs907715)*/*IRF5*-1/1 | 222 | 171 | **8.13×10^6^** | 2.03 (1.49-2.78) |
| *IL21(rs907715)*/*IKZF1*-0/0 | 17 | 21 |  | 1.00 |
| *IL21(rs907715)*/*IKZF1*-0/1 | 132 | 193 | 0.63 | 0.85 (0.43-1.66) |
| *IL21(rs907715)*/*IKZF1*-1/0 | 40 | 79 | 0.21 | 0.63 (0.30-1.32) |
| *IL21(rs907715)*/*IKZF1*-1/1 | 669 | 674 | 0.54 | 1.23 (0.64-2.35) |
| *IL21(rs907715)*/*ETS1*-0/0 | 49 | 76 |  | 1.00 |
| *IL21(rs907715)*/*ETS1*-0/1 | 100 | 138 | 0.60 | 1.12 (0.72-1.75) |
| *IL21(rs907715)*/*ETS1*-1/0 | 240 | 347 | 0.73 | 1.07 (0.72-1.59) |
| *IL21(rs907715)*/*ETS1*-1/1 | 469 | 406 | **2.58×10^3^** | 1.79 (1.22-2.63) |
| *IL21(rs907715)*/*Mir146a*-0/0 | 95 | 145 |  | 1.00 |
| *IL21(rs907715)*/*Mir146a*-0/1 | 54 | 69 | 0.43 | 1.20 (0.77-1.86) |
| *IL21(rs907715)*/*Mir146a*-1/0 | 438 | 477 | 0.02 | 1.40 (1.05-1.87) |
| *IL21(rs907715)*/*Mir146a*-1/1 | 271 | 276 | 0.01 | 1.50 (1.10-2.04) |
| *IL21(rs2221903)* /*IRF5*-0/0 | 468 | 615 |  | 1.00 |
| *IL21(rs2221903)* /*IRF5*-0/1 | 202 | 175 | 5.03×10^4^ | 1.52 (1.20-1.92) |
| *IL21(rs2221903)* /*IRF5*-1/0 | 127 | 136 | 0.14 | 1.23 (0.94-1.61) |
| *IL21(rs2221903)* /*IRF5*-1/1 | 61 | 41 | **1.27×10^3^** | 1.96 (1.29-2.96) |
| *IL21(rs2221903)* /*IKZF1*-0/0 | 43 | 76 |  | 1.00 |
| *IL21(rs2221903)* /*IKZF1*-0/1 | 627 | 714 | 0.03 | 1.55 (1.05-2.29) |
| *IL21(rs2221903)* /*IKZF1*-1/0 | 14 | 24 | 0.94 | 1.03 (0.48-2.20) |
| *IL21(rs2221903)* /*IKZF1*-1/1 | 174 | 153 | **1.42×10^3^** | 2.01 (1.30-3.10) |
| *IL21(rs2221903)* /*ETS1*-0/0 | 233 | 341 |  | 1.00 |
| *IL21(rs2221903)* /*ETS1*-0/1 | 437 | 449 | 1.08**×**10^3^ | 1.42 (1.15-1.76) |
| *IL21(rs2221903)* /*ETS1*-1/0 | 56 | 82 | 1.00 | 1.00 (0.69-1.46) |
| *IL21(rs2221903)* /*ETS1*-1/1 | 132 | 95 | **6.91×10^6^** | 2.03 (1.49-2.78) |
| *IL21(rs2221903)* /*Mir146a*-0/0 | 414 | 514 |  | 1.00 |
| *IL21(rs2221903)* /*Mir146a*-0/1 | 256 | 276 | 0.20 | 1.15 (0.93-1.43) |
| *IL21(rs2221903)* /*Mir146a*-1/0 | 119 | 108 | 0.03 | 1.37 (1.02-1.83) |
| *IL21(rs2221903)* /*Mir146a*-1/1 | 69 | 69 | 0.24 | 1.24 (0.87-1.78) |
| *IRF5*/*IKZF1*-0/0 | 43 | 74 |  | 1.00 |
| *IRF5*/*IKZF1*-0/1 | 552 | 677 | 0.09 | 1.40 (0.95-2.08) |
| *IRF5*/*IKZF1*-1/0 | 14 | 26 | 0.84 | 0.93 (0.44-1.96) |
| *IRF5*/*IKZF1*-1/1 | 249 | 190 | **1.21×10^4^** | 2.26 (1.48-3.44) |
| *IRF5*/*ETS1*-0/0 | 193 | 319 |  | 1.00 |
| *IRF5*/*ETS1*-0/1 | 402 | 432 | 1.65**×**10^4^ | 1.54 (1.23-1.93) |
| *IRF5*/*ETS1*-1/0 | 96 | 104 | 0.01 | 1.53 (1.10-2.12) |
| *IRF5*/*ETS1*-1/1 | 167 | 112 | **2.23×10^9^** | 2.47 (1.83-3.32) |
| *IRF5*/*Mir146a*-0/0 | 365 | 482 |  | 1.00 |
| *IRF5*/*Mir146a*-0/1 | 230 | 269 | 0.29 | 1.13 (0.90-1.41) |
| *IRF5*/*Mir146a*-1/0 | 168 | 140 | 5.55**×**10^4^ | 1.59 (1.22-2.06) |
| *IRF5*/*Mir146a*-1/1 | 95 | 76 | 2.82**×**10^3^ | 1.65 (1.19-2.30) |
| *IKZF1*/*ETS1*-0/0 | 22 | 42 |  | 1.00 |
| *IKZF1*/*ETS1*-0/1 | 35 | 58 | 0.68 | 1.15 (0.59-2.24) |
| *IKZF1*/*ETS1*-1/0 | 267 | 381 | 0.29 | 1.34 (0.78-2.29) |
| *IKZF1*/*ETS1*-1/1 | 534 | 486 | **5.25×10^3^** | 2.10 (1.23-3.57) |
| *IKZF1*/*Mir146a*-0/0 | 35 | 64 |  | 1.00 |
| *IKZF1*/*Mir146a*-0/1 | 22 | 36 | 0.75 | 1.12 (0.57-2.19) |
| *IKZF1*/*Mir146a*-1/0 | 498 | 558 | 0.02 | 1.63 (1.06-2.51) |
| *IKZF1*/*Mir146a*-1/1 | 303 | 309 | 8.88**×**10^3^ | 1.79 (1.15-2.79) |
| *ETS1*/*Mir146a*-0/0 | 181 | 274 |  | 1.00 |
| *ETS1*/*Mir146a*-0/1 | 108 | 149 | 0.56 | 1.10 (0.80-1.50) |
| *ETS1*/*Mir146a*-1/0 | 352 | 348 | 4.66**×**10^4^ | 1.53 (1.21-1.95) |
| *ETS1*/*Mir146a*-1/1 | 217 | 196 | 1.64**×**10^4^ | 1.68 (1.28-2.19) |

Genotype combinations were conducted under the dominant model.

*IL21* (rs907715) 0/1: AA/AG+GG; *IL21* (rs2221903) 0/1: AA/AG+GG; *IRF5* 0/1: GG/GA+AA; *IKZF1* 0/1: GG/GT+TT; *ETS1* 0/1: GG/GA+AA and *MiR146a* 0/1: AA/AG+GG.

Interactions were conducted by direct counting and chi-square tests using a 2×2 factorial design to calculate the attributable proportion due to interaction (AP) and the relative excess risk due to interaction (RERI).
