# Supplementary material for: Gene–Gene and Gene-Sex Epistatic Interactions of MiR146a, IRF5, IKZF1, ETS1 and IL21 in Systemic Lupus Erythematosus
Source: PLoS One. 2012 Dec 7;7(12):e51090. doi: 10.1371/journal.pone.0051090 (PMC3517573; doi:10.1371/journal.pone.0051090)
Supplement: Table S3 — Interaction analysis of gene-sex involved in systemic lupus erythematosus, by logistic regression. (DOC) [file pone.0051090.s005.doc]

**Table S3. Interaction analysis of gene-sex involved in systemic lupus erythematosus, by logistic regression***

| Gene | Codominant | Dominant | Recessive |
| --- | --- | --- | --- |
| *IL21*(rs907715) | 0.39 | **0.03** | 0.58 |
| *IL21*(rs2221903) | 0.14 | 0.59 | 0.75 |
| *IRF5* | 0.37 | 0.33 | 0.16 |
| *IKZF1* | 0.42 | 0.40 | 0.62 |
| *ETS1* | 0.60 | 0.30 | 0.27 |
| *MiR146a* | 0.10 | 0.16 | 0.21 |

*The data are presented as P values for departure from a multiplicative interaction model, obtained by log-likelihood ratio tests between the models, with and without an interaction term. An interaction term was considered significant if P< 0.05. The regression models were adjusted for age.
