# Supplementary material for: Gene–Gene and Gene-Sex Epistatic Interactions of MiR146a, IRF5, IKZF1, ETS1 and IL21 in Systemic Lupus Erythematosus
Source: PLoS One. 2012 Dec 7;7(12):e51090. doi: 10.1371/journal.pone.0051090 (PMC3517573; doi:10.1371/journal.pone.0051090)
Supplement: Table S4 — Sex–gene disparities between men and women with systemic lupus erythematosus. (DOC) [file pone.0051090.s006.doc]

**Table S4. Sex–gene disparities between men and women with systemic lupus erythematosus.**

| **Gene** | **SNP** | **Allele^a^** | **Male Case (MAF)** | **Female case (MAF)** | **OR (95% CI)** | **P-value** |
| --- | --- | --- | --- | --- | --- | --- |
| *IL21* | rs907715 | A/G | 0.420 | 0.420 | 1.00 (0.73-1.37) | 0.99 |
| *IL21* | rs2221903 | G/A | 0.119 | 0.114 | 1.05 (0.65-1.70) | 0.84 |
| *IRF5* | rs4728142 | A/G | 0.170 | 0.163 | 1.06 (0.70-1.60) | 0.80 |
| *IKZF1* | rs4917014 | G/T | 0.290 | 0.264 | 1.14 (0.81-1.60) | 0.47 |
| *ETS1* | rs6590330 | A/G | 0.364 | 0.412 | 0.82 (0.59-1.13) | 0.22 |
| *MiR146a* | rs57095329 | G/A | 0.176 | 0.218 | 0.77 (0.51-1.15) | 0.20 |

^a^ Minor allele/major allele; MAF, minor allele frequency; 95% CI, 95% confidence intervals.
