# Supplementary material for: Gene–Gene and Gene-Sex Epistatic Interactions of MiR146a, IRF5, IKZF1, ETS1 and IL21 in Systemic Lupus Erythematosus
Source: PLoS One. 2012 Dec 7;7(12):e51090. doi: 10.1371/journal.pone.0051090 (PMC3517573; doi:10.1371/journal.pone.0051090)
Supplement: Table S5 — Analysis of over- and under-sample data for gene-gene interaction. (DOC) [file pone.0051090.s007.doc]

**Table S5. Analysis of over- and under-sample data for gene-gene interaction.**

| **Model** | **Training Bal. Acc. (%)** | **Testing Bal. Acc. (%)** | **Cross-validation Consistency** |
| --- | --- | --- | --- |
| Over-sampled data |  |  |  |
| *ETS1* | 54.99 | 52.48 | 6/10 |
| *IL21(rs907715), ETS1* | 56.40 | 54.34 | 6/10 |
| *IL21(rs907715), IKZF1,ETS1* | 58.59 | 56.41 | 9/10 |
| ***IL21(rs907715),IRF5, IKZF1,ETS1*** | **60.31** | **56.88** | **10/10** |
| Under-sampled data |  |  |  |
| *ETS1* | 55.10 | 53.09 | 7/10 |
| *IL21(rs907715), IKZF1* | 56.51 | 54.43 | 4/10 |
| *IL21(rs907715), IKZF1,ETS1* | 58.90 | 55.71 | 8/10 |
| ***IL21(rs907715),IRF5, IKZF1,ETS1*** | **60.78** | **56.41** | **9/10** |
