# Supplementary material for: Gene–Gene and Gene-Sex Epistatic Interactions of MiR146a, IRF5, IKZF1, ETS1 and IL21 in Systemic Lupus Erythematosus
Source: PLoS One. 2012 Dec 7;7(12):e51090. doi: 10.1371/journal.pone.0051090 (PMC3517573; doi:10.1371/journal.pone.0051090)
Supplement: Table S6 — Analysis of over- and under-sampled data for gene-sex interaction. (DOC) [file pone.0051090.s008.doc]

**Table S6. Analysis of over- and under-sampled data for gene-sex interaction**

| **Model** | **Training Bal. Acc. (%)** | **Testing Bal. Acc. (%)** | **Cross-validation Consistency** |
| --- | --- | --- | --- |
| Over-sampled data |  |  |  |
| *Sex* | 60.60 | 60.60 | 10/10 |
| *Sex, IL21(rs907715)* | 61.28 | 59.88 | 5/10 |
| ***Sex,IRF5, ETS1*** | **62.83** | **62.62** | **10/10** |
| *Sex,* *IKZF1,ETS1, IL21(rs907715)* | 63.60 | 60.03 | 5/10 |
| Under-sampled data |  |  |  |
| *Sex* | 60.43 | 60.43 | 10/10 |
| *Sex, IKZF1* | 61.12 | 58.51 | 6/10 |
| ***Sex,IRF5, ETS1*** | **62.49** | **62.30** | **10/10** |
| *Sex,* *IKZF1,ETS1, IL21(rs907715)* | 63.46 | 59.27 | 4/10 |
