# Supplementary material for: Gene–Gene and Gene-Sex Epistatic Interactions of MiR146a, IRF5, IKZF1, ETS1 and IL21 in Systemic Lupus Erythematosus
Source: PLoS One. 2012 Dec 7;7(12):e51090. doi: 10.1371/journal.pone.0051090 (PMC3517573; doi:10.1371/journal.pone.0051090)
Supplement: Table S7 — Three-way gene-sex interactions of MDR analysis. (DOC) [file pone.0051090.s009.doc]

**Table S7. Three-way gene-sex interactions of MDR analysis.**

| **Rank^a^** | **Model** | **Training Bal. Acc. (%)** | **P-value*** |
| --- | --- | --- | --- |
| 1 | *Sex,IRF5, ETS1* | 62.61 | <0.001 |
| 2 | *Sex, ETS1, IKZF1* | 62.18 | <0.001 |
| 3 | *Sex, IL21(907715), IRF5* | 61.70 | <0.001 |
| 4 | *Sex, IL21(907715), IKZF1* | 61.67 | <0.001 |
| 5 | *Sex, IKZF1, IRF5* | 61.41 | <0.001 |

*Three-way interactions were validated based on 1000 permutations.

^a^ The rank was determined by the training-balanced accuracy of MDR.
